# The Relationship between Glycated Hemoglobin and Complexity of Coronary Artery Lesions among Older Patients with Diabetes Mellitus

**DOI:** 10.1371/journal.pone.0091972

**Published:** 2014-03-21

**Authors:** Jinling Ma, Xiujie Wang, Yutang Wang, Yuexiang Zhao, Meng Gao, Xiaoqian Li

**Affiliations:** 1 Department of Geriatric Cardiology, Chinese PLA General Hospital, Beijing, China; 2 Department of Radiology, Zhaoyuan People's Hospital, Shandong, China; Cardiff University, United Kingdom

## Abstract

**Objectives:**

Glycated hemoglobin (HbA1c) is associated with an increased risk of cardiovascular disease. The aim of this study was to examine the relationship between HbA1c levels and the complexity of coronary artery lesions among the older patients with diabetes mellitus (DM).

**Methods:**

This retrospective study enrolled a total of 3805 consecutive type 2 DM patients aged 60 years and older who underwent their first elective coronary angiography and had their HbA1c levels measured at the Chinese PLA General Hospital between December 2005 and December 2012.The complexity of the coronary artery lesions was evaluated using the Syntax score, and the subjects were divided into three groups according to their HbA1c levels. Logistic regression and Pearson correlation were used to analyze the association between the measured HbA1c levels and Syntax score.

**Results:**

The mean age was 72.3±10.6 years. The higher HbA1c levels were significantly associated with higher Syntax score (*p*<0.001). The unadjusted correlation coefficient of HbA1c levels and the Syntax score was 0. 371 (*p*<0.001). In addition, the higher HbA1c categories were able to independently predict patients with intermediate or high Syntax score (Syntax score ≥23) after adjustment for age, sex, hypertension, smoking, dyslipidemia and creatinine levels in the logistic regression analysis.

**Conclusion:**

HbA1c is significantly associated with the complexity of coronary lesions among older patients with DM. A higher HbA1c value is an independent predictor of the prevalence of complex coronary lesions. Further prospective multi-centre studies are needed to confirm this finding.

## Introduction

Diabetes mellitus (DM) is an important risk factor for coronary heart disease [1], and it is associated with a high prevalence of coronary artery disease (CAD) and an unfavorable prognosis [2]. Glycated hemoglobin (HbA1c) reflects the average blood glucose concentrations over the preceding 2 to 3 months [3]. Compared with the fasting blood glucose test, HbA1c has several advantages: it has higher repeatability [3], [4], can be assessed in the non-fasting state, and the committee highlighted that HbA1c is a more convenient test, with less biological variability and greater stability [5]. HbA1c levels may be of prognostic value with regard to future cardiovascular disease [6]. Several previous studies have demonstrated positive correlations of HbA1c with mortality and even subclinical cardiovascular disease in subjects without a history of diabetes [7]–[10]. Selvin et al. demonstrated that the HbA1c was also a strong predictor of future DM, cardiovascular disease, and all cause of mortality [11]. Moreover, DM is a major health problem for the aging population. Ageing is another factor that contributes to variance in the HbA1c and diabetes risk [12]. In elderly patients, the fear of iatrogenic hypoglycemia makes achievement of optimal glycemic control and HbA1c levels complex and generally only partially successful.

The Syntax score is an angiographic grading system based on the severity and complexity of the characteristics of coronary artery lesions [13], [14]. This system is widely accepted as a CAD complexity marker, and its prognostic value has been demonstrated in different clinical situations, and patients with the higher Syntax score have significantly more major adverse cardiac events (MACE) [15], [16].

The benefits of intensive therapy in an effort to lower HbA1c level should always be weighed against the greater risk of disabling and unpredictable hypoglycemia among older patients with DM, especially in elderly patients. No clinical trial data that exclusively represents the value of strict glycemic control in the geriatric population are available. Therefore, the aim of the present study was to evaluate the relationship between HbA1c levels and complexity of coronary artery lesions among older patients with DM.

## Methods

### Study population

We retrospectively reviewed the data of consecutive type 2 DM patients aged 60 years and older who underwent their first elective coronary angiography, to evaluate suspected CAD, at the Chinese PLA General Hospital between December 2005 and December 2012. Their medical history, lipid levels, creatinie levels, smoking status, demographic and angiographic data prior to undergoing coronary angiography were obtained from the hospital database. The indications for angiography in individuals in clinically stable condition were chest pain and/or noninvasive test results consistent with myocardial ischemia. We excluded patients whose HbA1c levels were not available during the hospitalization. The HbA1c of reference range for healthy nondiabetic individuals ranges from 4.1% to 6.5%. HbA1c was measured with an immunoassay (Bayer DCA-2000, Germany) during this time. Subjects with age <60 years, known type 1 DM, any previous history of coronary revascularization, or incomplete laboratory measurements were also excluded, resulting in a cohort of 3805 subjects for the present analysis. Patients were divided into three groups according to their HbA1c levels: 1535 patients with HbA1c levels of <6.4% (group 1), 1195 patients with HbA1c levels of 6.5% to 8.5% (group 2) and 1075 patients with HbA1c levels higher than 8.5% (group 3).

### Ethics Statement

The written informed consents were obtained from all subjects or their designated relatives. The Institutional Review Board of the PLA General Hospital approved this retrospective study.

### Syntax score and angiographic analysis

The severity of coronary artery lesions was quantified with the Syntax score. From the baseline diagnostic angiogram, each coronary lesion producing ≥50% diameter stenosis in vessels ≥1.5 mm was scored separately and added together to provide the overall Syntax score, which was calculated using the Syntax score algorithm[13], [14]. Coronary angiograms were analyzed by two experienced observers who were blinded to the identities and clinical information of the patients. Consistent with the SYNTAX trial, the low, intermediate and high Syntax score were defined as 0 to 22, 23 to 32 and 33 or more, respectively [17].

### Statistical analysis

The continuous variables are presented as the means ± SDs. The categorical variables are presented as proportions (percentages). Intergroup differences of continuous variables were analyzed by one-way ANOVA. Categorical variables were compared with χ^2^ tests. Linear regression analysis with Pearson's coefficient was used to assess the strength of association between HbA1c and Syntax score. Logistic regression analysis was used to predict the prevalence of an intermediate or high Syntax score, with adjustment in the age, sex, hypertension, smoking, dyslipidemia, and creatinine levels. All data were processed using the PASW (version 18.0; SPSS, Chicago, IL). A *p*-value <0.05 was considered to be significant.

## Results

**Figure 1:**
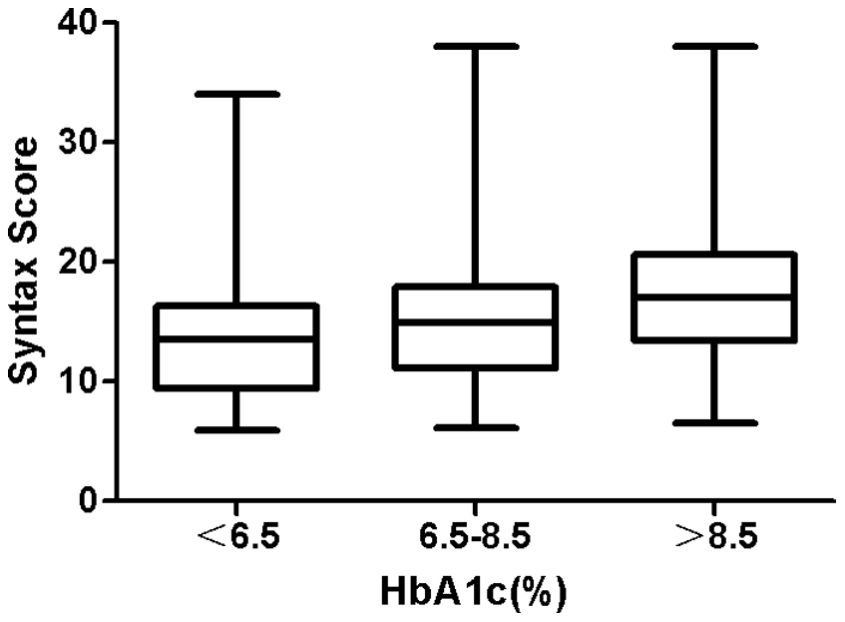
Syntax score according to HbA1c levels in type 2 DM patients aged 60 years and older.

Of the 3805 patients included in this study population, the mean age was 72.3±10.6 years. The subjects were divided into three groups: group I (HbA1c <6.5%); group II (6.5%≤HbA1c≤8.5%); and group III (HbA1c>8.5%). Baseline characteristics and laboratory data of the patients are summarized in Table 1. There were no significant differences in the proportion of male, sex, smoking and creatinine found among the three groups. In contrast, Syntax score was significantly different among the HbA1c groups, *p*<0.001(Fig 1). In addition, the higher HbA1c category was significantly associated with age, a higher proportion of male sex and hypertention, higher low density lipoprotein (LDL) cholesterol and triglyceride, and lower high density lipoprotein (HDL) cholesterol.

**Table 1:** Baseline characteristics and laboratory data of the type 2 DM patients aged 60 years and older.

	HbA1c Groups			
	I(<6.5%)	II (6.5%≤HbA1c≤8.5%)	III (>8.5%)	*p* Value
	n = 1535	n = 1195	n = 1075	
Age,years (mean±SD)	66.4±7.9	73.3±10.2	79.4±10.5	<0.001
Sex,male/female	862/673	643/552	468/407	0.3299
Smoking,%	38.6	34.9	36.3	0.1320
Hypertention,%	23.1	47.9	65.3	<0.001
Creatinine (mg/dl)	1.34±0.36	1.37±0.46	1.37± 0.53	0.129
LDL cholesterol (mg/dl)	107.9±9.9	113.1±10.6	117.2±10.3	<0.001
HDL cholesterol (mg/dl)	43.6±6.2	41.3±5.2	38.3±4.1	<0.001
Triglyceridea (mg/dl)	148.7±9.2	151.5±9.2	154.8±9.0	<0.001
Syntax score	13.3±4.3	14.8±4.9	17.4±5.7	<0.001

LDL: low density lipoprotein; HDL: high density lipoprotein.

**Figure 2:**
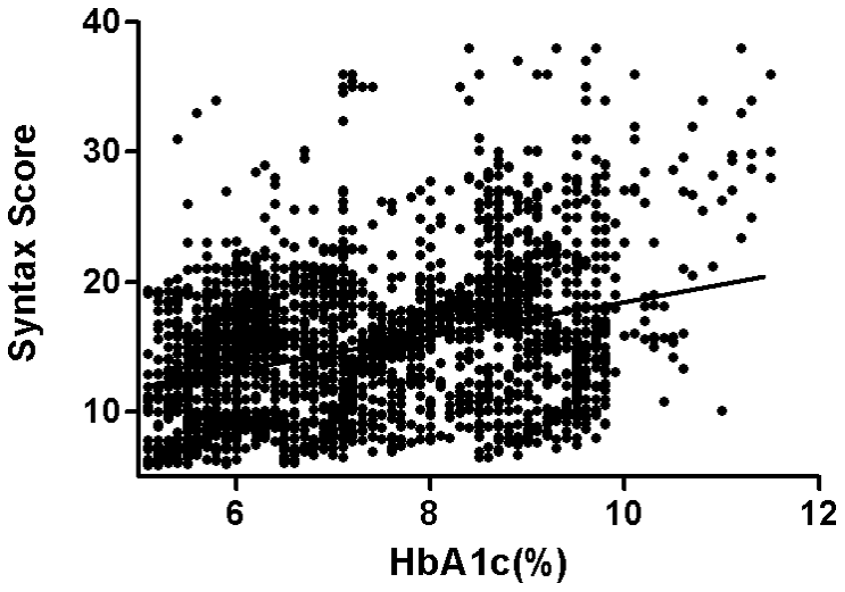
Correlation between HbA1c levels and Syntax score in type 2 DM patients aged 60 years and older.

The HbA1c levels and Syntax score were correlated (Fig 2) (r = 0.371; *p*<0.001). Moreover, the higher HbA1c categories were able to independently predict patients with intermediate or high Syntax score (Syntax score≥23) after the age, sex, hypertension, smoking, dyslipidemia, and creatinine levels were adjusted in the logistic regression analysis (Table 2).

**Table 2:** Adjusted odds ratios for prediction of high Syntax score patients.

Risk factors	Adjusted Odds Ratio	*p* Value
	(95% Confidence Interval)	
Age, years	1.03(1.013–1.049)	0.0005
Sex, male/female	0.92(0.689–1.240)	0.598
Smoking,%	1.00(0.736–1.354)	0.9907
Hypertention,%	0.91(0.599–1.381)	0.6577
Creatinine (mg/dl)	0.89(0.672–1.175)	0.406
LDL cholestorol (mg/dl)	0.98(0.965–0.999)	0.0416
HDL cholestorol (mg/dl)	1.00(0.965–1.037)	0.9847
Triglyceridea (mg/dl)	0.98(0.956–1.011)	0.2234
HbA1c,%	4.95(3.625–6.757)	0.0000

LDL: low density lipoprotein; HDL: high density lipoprotein.

## Discussion

DM has been associated with the extent of coronary atherosclerosis measured by coronary angiography and coronary multi-detector computed tomography [18]–[20]. In light of the severity of CAD in diabetic patients, it is necessary to take measures to prevent or delay its occurrence and development. However, in older patients, the benefits of intensive therapy to lower HbA1c level must always be weighed against the greater risk of disabling and unpredictable hypoglycemia, considering that the geriatric population is less likely to benefit from reducing the risk of microvascular complications and more likely to suffer serious adverse effects from hypoglycemia [21].

The Syntax score, can be especially useful in deciding the optimal treatment strategy in patients with complex lesions [15]. In particular, it is a useful tool to guide decision-making in patients undergoing three-vessel disease and left main percutaneous coronary intervention [22]. Zhao et al. recommend the use the clinical SYNTAX score for routine clinical decision-making [23]. In addition, the Syntax score has been shown to be an independent predictor of mortality and MACE at long-term follow-up [15], [16], [24]–[30].HbA1c is a useful marker of cardiovascular risk, even more superior to fasting glucose for long-term macrovascular risk stratification [9].

The principal findings of our study indicate that HbA1c level is associated with coronary lesion complexity in older diabetic patients. Previous studies have found an association between elevated cardiovascular risk and elevated HbA1c level [7], [8], [31]–[37], independent of classical risk factors [38]. Chronic hyperglycemia is associated with an increased risk for cardiovascular outcomes and all-cause mortality among patients with type 2 diabetes [39]. Nishimura et al. suggested that the control of HbA1c, was necessary to reduce the cardiovascular risk in diabetic patients with elevated HbA1c [40]. However, some clinical trials have shown little benefit, and possibly some harm, of lowering the glycated hemoglobin value in patients with DM to prevent cardiovascular outcomes [41]–[46]. In addition, those studies did not investigate the coronary artery lesion morphology quantified with the Syntax score among the exclusive older DM patients. Therefore, the aim of the present study was to evaluate the relationship between HbA1c levels and complexity of coronary artery lesions among older patients with DM. Also, this retrospective, prevalence study is quite a stretch in view of the failure of large randomized trials to demonstrate a beneficial effect.

In the present study, we found that HbA1c is an independent predictor of the prevalence of complex coronary artery lesions (Syntax score ≥23) in older diabetic patients. We established that the HbA1c level is significantly associated with the complexity of coronary lesions, independent of age, sex, and other cardiovascular risk factors such as hypercholesterolemia, hypertension, and smoking. Our findings indicate that the severity of coronary artery lesions is also notably influenced by the HbA1c, irrespective of other cardiovascular risk factors, age, and gender. Even after adjustment for these factors, HbA1c was still significantly associated with Syntax score. Additionally, the multivariate regression analysis showed that HbA1c is independently associated with Syntax score. Our study therefore demonstrated that, among older DM patients, HbA1c values can be a predictor of the prevalence of complex coronary artery lesions. The patients with the lower HbA1c levels have a distinctly lower risk of complex coronary artery lesions. Therefore, diabetic patients with cardiovascular risk factors should pay more attention to their blood glucose levels and potential cardiovascular complications, even among older patients with DM. Furthermore, the relationship between the Syntax score and HbA1c demonstrated in this study supports using HbA1c as a simplified indicator of prognosis. This study suggest that an improvement in glycemic control results in more favorable intermediate or long-term clinical outcomes in diabetic patients aged 60 years and older. However, the relationship between glycemic control and coronary atherosclerosis is not a simple one. There are many unmeasured potentially important covariates that may play a role. Maybe, for example, the duration of the diabetic process leads to both worse coronary atherosclerosis and higher HbA1c. As reported in a study, they found that interwoven actions of poor glycemic control, low grade inflammation and low HDL-cholesterol on atherosclerotic processes in type 2 diabetes [47]. Therefore, further studies are necessary to investigate the relation between HbA1c and Syntax score among older diabetic patients.

There were several limitations in this study which should be considered. This study was a retrospective and non-randomized study. First, our patients are only those who underwent first elective coronary angiography. These patients comprise some patients with CAD and may not represent all patients with CAD. Second, several confounding factors may not have been properly accounted for in the analysis. Consequently, numerous patients were excluded, and selection bias may have affected the results. Third, the present study excluded patients with a history of PCI or CABG, therefore our findings may not apply to these excluded subjects who represent more severe cases. We also did not collect information on severe obesity, peripheral vascular disease, cerebrovascular disease, estimated glomerular filtration rate, or duration of diabetes, which were also associated with the burden of vascular atherosclerosis. Thus, we should interpret the results very carefully. Finally, our study is a single-center observational experience. Most of the patients came from Beijing and its neighboring areas, so their glucose metabolic state may not completely be in coincidence with population of other places. Further prospectively multiple centre studies are required to better quantify this finding. Although caution is necessary for the interpretation of our data, we consider it improbable that these limitations have influenced our main findings.

In conclusion, the HbA1c is significantly associated with the complexity of coronary lesions amongst type 2 DM patients aged 60 years and older. The present study reveals that higher HbA1c value is an independent predictor of the prevalence of complex coronary lesions, even after adjusting the age and other coronary atherosclerotic risk factors. HbA1c may be a useful indicator of type 2 DM in patients aged 60 years and older with greatest absolute risk of cardiovascular disease. HbA1c could be used as a simple prognostic value after primary PCI in coronary care unit, and it also helps for the strict control of complications and preventive strategies. Control of glycemic metabolism may play an important role in reducing the HbA1c levels. For patients without hypoglycemia, further study is necessary to evaluate the benefit of intensive treatment of glycometabolic disorder to reduce the HbA1c level.
